# Radiomics Profiling Identifies the Value of CT Features for the Preoperative Evaluation of Lymph Node Metastasis in Papillary Thyroid Carcinoma

**DOI:** 10.3390/diagnostics12051119

**Published:** 2022-04-29

**Authors:** Guoqiang Yang, Fan Yang, Fengyan Zhang, Xiaochun Wang, Yan Tan, Ying Qiao, Hui Zhang

**Affiliations:** 1 Department of Radiology, First Hospital of Shanxi Medical University, Taiyuan 030001, China; yangguoqiang@sxmu.edu.cn (G.Y.); zhangfy@sxmu.edu.cn (F.Z.); wangxch@sxmu.edu.cn (X.W.); tany@sxmu.edu.cn (Y.T.); 2 College of Medical Imaging, Shanxi Medical University, Taiyuan 030001, China; yangfan@b.sxmu.edu.cn

**Keywords:** computed tomography, radiomics, papillary thyroid carcinoma, cervical lymph node metastasis, nomogram

## Abstract

Background: The aim of this study was to identify the increased value of integrating computed tomography (CT) radiomics analysis with the radiologists’ diagnosis and clinical factors to preoperatively diagnose cervical lymph node metastasis (LNM) in papillary thyroid carcinoma (PTC) patients. Methods: A total of 178 PTC patients were randomly divided into a training (*n* = 125) and a test cohort (*n* = 53) with a 7:3 ratio. A total of 2553 radiomic features were extracted from noncontrast, arterial contrast-enhanced and venous contrast-enhanced CT images of each patient. Principal component analysis (PCA) and Pearson’s correlation coefficient (PCC) were used for feature selection. Logistic regression was employed to build clinical–radiological, radiomics and combined models. A nomogram was developed by combining the radiomics features, CT-reported lymph node status and clinical factors. Results: The radiomics model showed a predictive performance similar to that of the clinical–radiological model, with similar areas under the curve (AUC) and accuracy (ACC). The combined model showed an optimal predictive performance in both the training (AUC, 0.868; ACC, 86.83%) and test cohorts (AUC, 0.878; ACC, 83.02%). Decision curve analysis demonstrated that the combined model has good clinical application value. Conclusions: Embedding CT radiomics into the clinical diagnostic process improved the diagnostic accuracy. The developed nomogram provides a potential noninvasive tool for LNM evaluation in PTC patients.

## 1. Introduction

Papillary thyroid carcinoma (PTC) is the most common primary thyroid malignancy, occurring in 90% of patients with thyroid carcinoma [1,2]. The incidence of PTC has increased dramatically in recent decades [3]. Although PTC is an inert cancer with relatively low recurrence and mortality rates, metastasis remains a concerning clinical problem. The occurrence of cervical lymph node metastasis (LNM) in patients with PTC is highly associated with local recurrence and poor overall survival [4,5]. Cervical LNM can be divided into central LNM (level VI) and lateral LNM (level II-V). The revised American Thyroid Association (ATA) guidelines recommend ipsilateral lobectomy for small unifocal tumors without extrathyroidal extension or LNM because of the slow progression of PTC [6]. For patients with evident lateral LNM, therapeutic lateral neck lymph node dissection is recommended [7]. Therefore, an accurate preoperative identification of cervical LNM is important for optimal staging, individualized treatment planning and prognosis evaluation for patients with PTC.

Biopsy is the gold standard for preoperatively confirming LNM. Fine-needle aspiration (FNA) is widely considered the most accurate and cost-effective diagnostic method for evaluating LNM; however, it is invasive and time-consuming and puts patients at risk of infection [8,9]. Ultrasound (US) is currently the main noninvasive imaging modality for preoperatively evaluating LNM in PTC, with a reported high specificity (92.8%) and relatively low sensitivity (59.1%) [10]. However, the diagnostic accuracy of US is substantially affected by operator experience, and the technique has difficulty diagnosing LNM in the inferior cervical, retropharyngeal, retrosternal, and mediastinum spaces [11]. Magnetic resonance imaging (MRI) has high soft-tissue resolution and specificity in evaluating cervical LNM in PTC (91%) [12]; MRI also has advantages in evaluating large cervical lymph node lesions [13]. MRI showed high sensitivity (95%) and accuracy (83%) and relatively low specificity (51%) in detecting cervical LNM of differentiated thyroid cancer [14]. Positron emission tomography (PET)/computed tomography (CT) is extremely sensitive and specific in diagnosing cervical LNM [15,16,17]. At present, although CT is not the main imaging modality for diagnosing PTC, some studies have confirmed its advantages in the diagnosis of LNM in PTC patients [18,19]. Lee et al. found that CT showed better diagnostic values for cervical LNM than US in patients with papillary thyroid microcarcinoma whose tumor has characteristics suitable for active surveillance (AS) [20]. Nonetheless, in clinical practice, regardless of the modality used (US, MRI, PET/CT or CT), morphological characteristics or certain semiquantitative parameter values are typically used to diagnose LNM, but even this is limited by subjectivity and poor reproducibility.

Radiomics has recently emerged as a promising technique for cancer research; it is based on the hypothesis that the quantitative analysis of medical imaging can capture additional information to describe tumor heterogeneity [21,22]. Radiomics relies on the assumption that medical images contain biological or medical data information of lesions that cannot be recognized by the naked eye [23]. The core principle of radiomics is to use high-throughput algorithm to extract massive features from tumor ROI and use the information that cannot be recognized by the naked eye for diversified statistical analysis, which can be finally used for clinical disease prediction and auxiliary diagnosis [24]. More recently, radiomics has been widely applied in the clinical decision making for many cancers to predict clinical stage and pathological type, gene expression, treatment responses and prognosis [25,26,27,28]. In addition, radiomics has been used to preoperatively predict LNM in many cancers, such as colorectal cancer [29], breast cancer [30], lung cancer [31], gastric cancer [32] and biliary tract cancer [33]. For predicting LNM in PTC, there are many radiomic studies based on US images [34,35,36], but relatively few studies have investigated CT radiomics. Lu et al. found that the radiomics analysis based on noncontrast and venous contrast-enhanced CT images improved the preoperative prediction of cervical LNM in PTC patients [37]. Zhou et al. found that the radiomics analysis of dual-energy CT-derived iodine maps showed better diagnostic performance than qualitative evaluations of CT signs for predicting cervical LNM in PTC patients [38].

The aim of our study was to develop and validate a combined nomogram integrating CT radiomics, classic imaging features and clinical factors to preoperatively predict cervical LNM in patients with PTC and to identify the increased value of embedding CT radiomics into the clinical diagnostic process to improve diagnostic accuracy.

## 2. Materials and Methods

### 2.1. Patients

**Figure 1:**
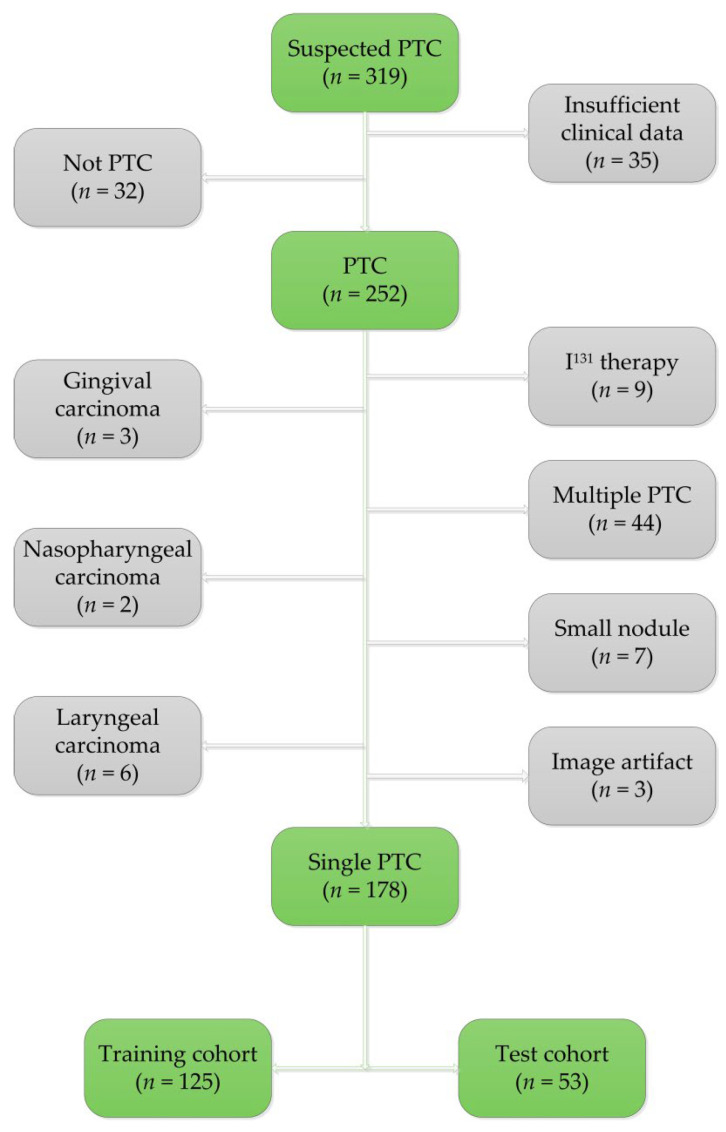
Exclusion criteria and details of the patients in the training and test cohorts.

This retrospective study was approved by the institutional review board of the First Hospital of Shanxi Medical University (2021-K-K140), and carried out in accordance with the Declaration of Helsinki. The requirement for informed consent was signed for all patients. A total of 319 patients with suspected PTC in our institution from December 2017 to April 2021 were collected and screened. All patients identified in this study met the following inclusion criteria: (1) pathologically confirmed to have PTC; (2) a pathological lymph node diagnosis; (3) preoperative noncontrast CT scan and dual-phase dynamic contrast-enhanced CT images of sufficiently high quality for analysis; and (4) sufficient available clinical information. The exclusion criteria were as follows: (1) preoperative therapy (such as radiotherapy, chemotherapy or interventional therapy); (2) other malignancies; (3) postoperative pathological confirmation of multifocal PTC; and (4) unclear CT images of the primary tumors or tumors too small for segmentation and analysis (maximum diameter ≤ 3 mm). Finally, 178 patients (43 males and 135 females) with a mean age of 45.57 ± 13.00 years (range 9 to 73 years) who met the inclusion and exclusion criteria were enrolled in the study. The details of the included and excluded patients are shown in Figure 1. A total of 178 patients with PTC were divided into two groups: LNM (+) (*n* = 100) and LNM (−) (*n* = 78). Chi-square tests compared the clinical and radiological parameters between the LNM (+) and LNM (−) groups. The clinical and radiological factors with statistical difference were identified as the most diagnostic clinical and radiological factors. Clinical information, including age, sex, body mass index (BMI), thyroid hormone level and presence of nodular goiters or Hashimoto’s thyroiditis, was obtained through the medical records system. According to clinical experience, the normal standards for BMI, thyroglobulin (TG), thyroid-stimulating hormone (TSH), free triiodothyronine (FT3), free thyroxine (FT4), thyroglobulin antibody (TGAb) and thyroid peroxidase antibody (TPOAb) were as follows: BMI, 18.5–23.9; TG, 3.5–77 ng/mL; TSH, 0.27–4.2 uIU/mL; FT3, 3.1–6.8 pmol/L; FT4, 12–22 pmol/L; TGAb, 0–115 IU/mL; TPOAb, 0–34 IU/mL. Any values outside these ranges were considered abnormal. Then, according to the age and sex listed in the clinical data, the patients were randomly divided into a training cohort (*n* = 125) and test cohort (*n* = 53) with a ratio of 7:3.

### 2.2. CT Acquisition

Preoperative noncontrast and dual-phase contrast-enhanced CT scanning was performed with a third-generation Siemens dual-source CT device (Somatom Definition, Siemens, Germany) or a Philips spectral CT device (IQon spectral CT, Philips, The Netherlands). The patients were placed in a supine head-first position. The scanning range was from the skull base to the upper edge of the aortic arch. The patients were instructed not to swallow during scanning and to drop their shoulders as much as possible to avoid swallowing and clavicle artifacts. The CT parameters were as follows: (1) A and B X-ray tube voltages of 90 kV and Sn150 kV, respectively; automatic tube current regulation technology; pitch factor, 1.5; rotation time, 0.25 s; detector collimation, 192 × 0.6 mm; slice thickness, 2 mm; and reconstruction slice thickness, 0.75 mm; (2) tube voltage, 120 kV; tube current, 50–350 mAs; pitch factor, 0.969; rotation time, 0.5 s; detector collimation, 64 × 0.625 mm; slice thickness, 2 mm; and reconstruction slice thickness, 0.75 mm. After routine noncontrast CT scanning, 60 mL of iodinated nonionic contrast medium (iodixanol injection, Hengrui Medicine, China) was injected through the cubital vein at a flow rate of 3.5 mL/s with a concentration of 320 mg I/mL. Then, the patients underwent contrast-enhanced scanning 25 s (arterial phase) and 50 s (venous phase) after injection.

### 2.3. Radiologist Assessment of the Primary Tumors and LNMs

For the morphological evaluation of the primary tumors, two radiologists (5 and 7 years of diagnostic experience) observed and recorded the location of the primary PTC tumor (right lobe, left lobe or isthmus) and measured the anteroposterior diameter (AD), transverse diameter (TD), anteroposterior to transverse diameter ratio (A/T) and degree of calcification and capsule invasion. In cases of disagreement on qualitative indicators, a consensus was reached through discussion. The mean value of the quantitative indicators was taken as the final value. The anteroposterior and transverse diameters were recorded as greater or less than 6 mm. The A/T ratio was recorded as greater or less than 1 [39].

**Figure 2:**
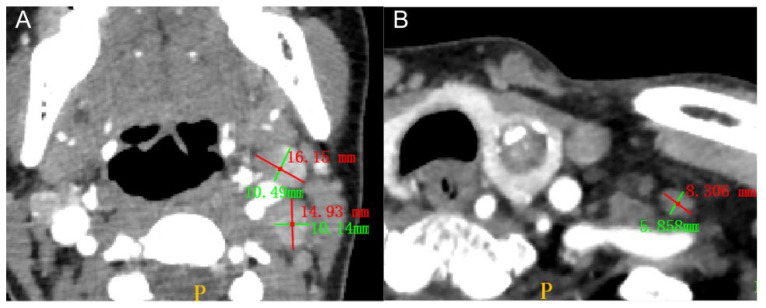
(**A**) Two metastatic lymph nodes, demonstrating substantial but uneven enhancement in the arterial phase, with maximum short-axis diameters of more than 10 mm (maximum long-axis diameters of 16.15 mm and 14.93 mm and maximum short-axis diameters of 10.49 mm and 10.14 mm, respectively). (**B**) A normal, oval and nonmetastatic lymph node, seen to the left of the primary lesion (maximum long-axis diameter of 8.31 mm and maximum short-axis diameter of 5.86 mm).

Three radiologists with 15, 7 and 5 years of diagnostic experience in head and neck cancers independently and blindly assessed lymph node status, and their diagnosis results are referred to as CT-reported LN status 1, CT-reported LN status 2 and CT-reported LN status 3, respectively. According to the National Comprehensive Cancer Network (NCCN) guidelines and previous studies [40,41], LNM was considered for patients who met at least one of the following conditions: (1) lymph node size larger than 10 mm; (2) round or irregular shape; (3) obscure boundary or encroachment of adjacent tissues; (4) calcified, cystic or necrotic tissue; (5) obvious enhancement; and (6) inhomogeneous enhancement. Figure 2 shows a typical case of LNM and one without LNM on CT.

### 2.4. Tumor Segmentation and Feature Extraction

**Figure 3:**
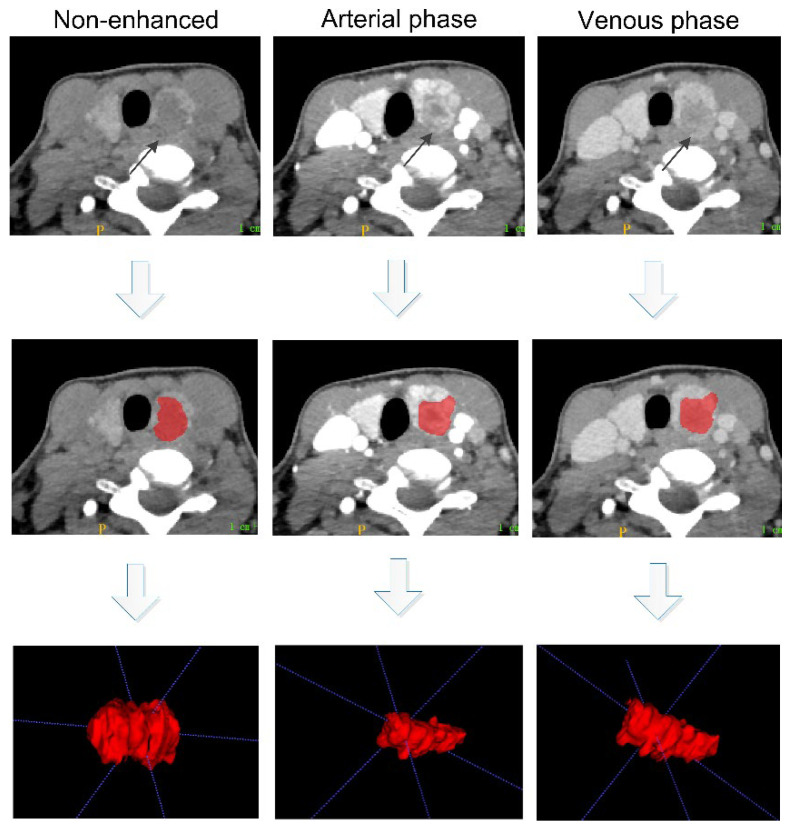
Segmentation of the ROIs on three-phase (nonenhanced, arterial phase and venous phase) axial CT images. Three-dimensional reconstruction was performed using the ROIs from the axial, sagittal and coronal plane images. The black arrows indicate the location of the primary lesion. Abbreviation: ROIs, regions of interest.

Manual segmentation was performed slice by slice on the unenhanced CT images and arterial and venous contrast-enhanced CT images by a radiologist with 15 years of diagnostic experience using ITK-SNAP (www.itksnap.org accessed on 25 April 2022) to obtain the tumor regions of interest (ROIs). The three-phase CT images were delineated with a constant window width (350 HU) and window level (60 HU) without avoiding cystic areas, areas of necrosis, hemorrhage and calcification. The following guideline was followed when performing ROI segmentation: the three-phase CT images were compared when the boundary of the lesion was not clear, and areas with the same enhancement pattern were included in the ROI when the lesion invaded the surrounding tissue. Figure 3 shows the 3D ROI segmentation of primary PTC tumors on three-phase CT images.

After tumor segmentation, radiomic feature extraction was automatically performed within the ROI on each of the three-phase CT images using FeAture Explorer (FAE) software [42], based on the Pyradiomics open source module [43]. A total of 851 features were extracted from the ROI in each phase, including 18 first-order, 14 shape, 24 gray-level cooccurrence matrix (GLCM), 16 gray-level run-length matrix (GLRLM), 16 gray-level size zone matrix (GLSZM), 5 neighborhood gray-tone difference matrix (NGTDM), 14 gray-level dependent matrix (GLDM) and 744 wavelet features. Finally, a total of 2553 features were extracted from the three-phase CT images of each patient. To evaluate the stability and reproducibility of the radiomics features, 20 patients were randomly selected for a double-blinded comparison of the manual segmentations by two radiologists, and features with intraclass and interclass correlation coefficients (ICCs) lower than 0.75 were removed for further analysis.

### 2.5. Feature Selection and Model Construction

After the stability and reproducibility evaluations, the remaining features were normalized by transforming the data into new scores using mean or z-score normalization (with a mean of 0 and a standard deviation of 1). We chose principal component analysis (PCA) or Pearson’s correlation coefficient (PCC) feature dimension reduction methods to remove redundant features, and then ANOVA or Kruskal–Wallis test methods were used to select the most useful predictive LNM-related features.

We constructed three clinical–radiological models based on the clinical factors and CT-reported lymph node status assessed by three radiologists with 5, 7 and 15 years of diagnostic experience. Univariable analyses were used to identify the clinical–radiological factors associated with LNMs. A noncontrast radiomics model, arterial contrast radiomics model and venous contrast radiomics model were constructed based on the corresponding selected feature subsets, and then a three-phase radiomics model was constructed based on the selected features of all three feature subsets. Radiomics scores (Rad scores) were calculated using a linear combination of the final selected features with their corresponding weights to build a radiomics signature. Finally, a combined model integrating the most diagnostic clinical factors, radiological characteristics and the radiomics signature was constructed. All models were developed using multiple logistic regression algorithms. We used internal 5-fold cross-validation on the training cohort for hyperparameter optimization and to train the optimal model. The predictive performances of the different models were evaluated using receiver operating characteristic (ROC) curve analysis, quantified by area under the curve (AUC) and accuracy (ACC), and compared through the DeLong test. The feature selection and model construction were performed using FAE software, which provides a radiomics pipeline to develop radiomic models with different combinations of feature normalization methods, feature selection algorithms and classifiers [41]. The best model configuration was determined based on the highest AUC value in the test cohort.

A nomogram for the combined model was generated to provide clinicians with an individualized and visual tool for diagnosing LNM in PTC. The clinical and radiological parameters were identified in the nomogram model by using binary logistic regression analysis, and the forward selection method was used to screen the variables to construct the nomogram as potential predictors (enter value: 0.05, remove value: 0.10). To evaluate the agreement between the nomogram-predicted and actual LNM probabilities, calibration curves were plotted, and the Hosmer–Lemeshow test was applied in both the training cohort and test cohort. Decision curve analysis was performed to compare the combined model, the three-phase radiomics model and the clinical–radiological model in terms of clinical usefulness by quantifying the net benefits at different threshold probabilities in the test cohort.

### 2.6. Statistical Analysis

Feature extraction, feature selection and model construction were performed with FAE software (https://github.com/salan668/FAE accessed on 25 April 2022). We performed the statistical analysis with SPSS software (version 23.0) and R software (version 3.6.1, https://www.r-project.org accessed on 25 April 2022). For continuous variables, Student’s t-test or the Kruskal–Wallis test was used to assess the statistical significance of differences between groups. For categorical variables, Pearson’s chi-square test was used. A two-sided *p*-value less than 0.05 was considered statistically significant.

## 3. Results

### 3.1. Patient Characteristics and the Clinical–Radiological Models

The characteristics of the PTC patients in the LNM (+) and LNM (−) groups are shown in Table 1. Age, capsule, AD, TD, A/T, CT-reported LN status 1 and CT-reported LN status 3 were significantly different between the two groups (*p* < 0.05). Table 2 shows the characteristics of the patients in the training cohort and test cohort. There were no significant differences between the two cohorts in any of the clinical, radiological and pathological characteristics (all *p* > 0.05). Table 3 shows the association between the actual LNM state and patient characteristics in the training cohort and test cohort. Clinical–radiological model 1 was established with two features and achieved an AUC of 0.781 in the training cohort and 0.758 in the test cohort. Clinical–radiological model 2 was established with seven features and achieved an AUC of 0.796 in the training cohort and 0.729 in the test cohort, and clinical–radiological model 3 was established with six features and achieved an AUC of 0.800 in the training cohort and 0.743 in the test cohort. The coefficients of the features in the clinical–radiological models are shown in Supplementary Table S1.

### 3.2. Feature Selection and Radiomic Models

Most of the radiomics features that showed favorable stability and reproducibility (ICC > 0.75) in the intraobserver and interobserver agreement assessment were selected for further analysis, including 794 (93.9%) features from the noncontrast CT images, 770 (90.5%) features from the arterial contrast-enhanced CT images and 778 (91.2%) features from the venous contrast-enhanced CT images. After feature reduction and selection, 16 features were selected to construct the noncontrast model, 15 were selected to construct the arterial contrast model and 11 were selected to construct the venous contrast model. Of the combined 42 features of the three models, 14 were further selected by PCC and ANOVA to construct the three-phase radiomics model using multiple logistic regression. After integrating the most diagnostic clinical factors, radiological characteristics and radiomics features, 10 features were selected by PCA and ANOVA methods to construct the combined model using multiple logistic regression. The coefficients of the selected radiomics features in the different models are shown in Supplementary Table S2.

The predictive performances of the different models are shown in Table 4. The noncontrast model yielded an AUC, ACC, sensitivity and specificity of 0.786, 72.80%, 84.29% and 58.18%, respectively, in predicting LNM in the training cohort and 0.781, 73.58%, 80.00% and 65.22%, respectively, in the test cohort. The arterial contrast model yielded an AUC, ACC, sensitivity and specificity of 0.808, 74.40%, 71.439% and 78.18%, respectively, in the training cohort and 0.791, 75.47%, 66.67% and 86.96%, respectively, in the test cohort. The venous contrast model yielded an AUC, ACC, sensitivity and specificity of 0.827, 76.80%, 87.14% and 63.64%, respectively, in the training cohort and 0.790, 75.47%, 86.67% and 60.87%, respectively, in the test cohort. The three-phase radiomics model yielded an AUC, ACC, sensitivity and specificity of 0.790, 72.80%, 78.57% and 65.458%, respectively, in the training cohort and 0.813, 79.25%, 80.00% and 78.26%, respectively, in the test cohort. The three-phase radiomics model yielded a similar diagnostic performance to clinical–radiological model 1 (clinical–radiological factors combined with the CT report from the radiologist with 15 years of diagnostic experience) in the training cohort (AUC, 0.790 vs. 0.781, DeLong test *p* = 0.863; ACC, 72.8% vs. 75.2%) and test cohort (AUC, 0.813 vs. 0.758, DeLong test *p* = 0.483; ACC, 79.25% vs. 73.58%). When the most diagnostic clinical factors, radiological characteristics and radiomics features were combined, the resulting model yielded the best predictive performance with an AUC, ACC, sensitivity, and specificity of 0.868, 86.83%, 88.57% and 70.91%, respectively, in the training cohort and 0.878, 83.02%, 90.00% and 73.91%, respectively, in the test cohort.

In order to evaluate the diagnostic performance of the combined model in different LNM location subgroups, all of the 100 patients with LNM were divided into three groups: central LNM (33 cases), lateral LNM (27 cases) and central and lateral LNM (40 cases). The diagnostic performance of the combined model was evaluated though a stratified analysis in the central LNM, lateral LNM and central and lateral LNM subgroups. To ensure the balance of the dataset, we randomly selected 33 patients, 27 patients and 40 patients in the non-metastatic group (78 cases), respectively, as the control groups. Table 5 shows the diagnostic performance of the combined model in different LN location subgroups. The combined model has a certain diagnostic value in predicting central LNM, lateral LNM and central and lateral LNM for PTC patients. The AUC of the combined model in the central LNM prediction (AUC = 0.833) was similar to that of the lateral LNM prediction (AUC = 0.823). However, the sensitivity of the combined model in the central LNM prediction (78.79%) is higher than that of the lateral LNM prediction (66.67%), and the specificity of the combined model in the central LNM prediction (72.73%) is relatively lower than that of the lateral LNM prediction (81.48%). Each parameter of the combined model in the central and lateral LNM prediction is more than 85%, and the AUC is as high as 0.960. The combined model had the most sensitive and specific for diagnosing patients with central and lateral LNM.

### 3.3. Radiomics Nomogram and Clinical Utility

**Figure 4:**
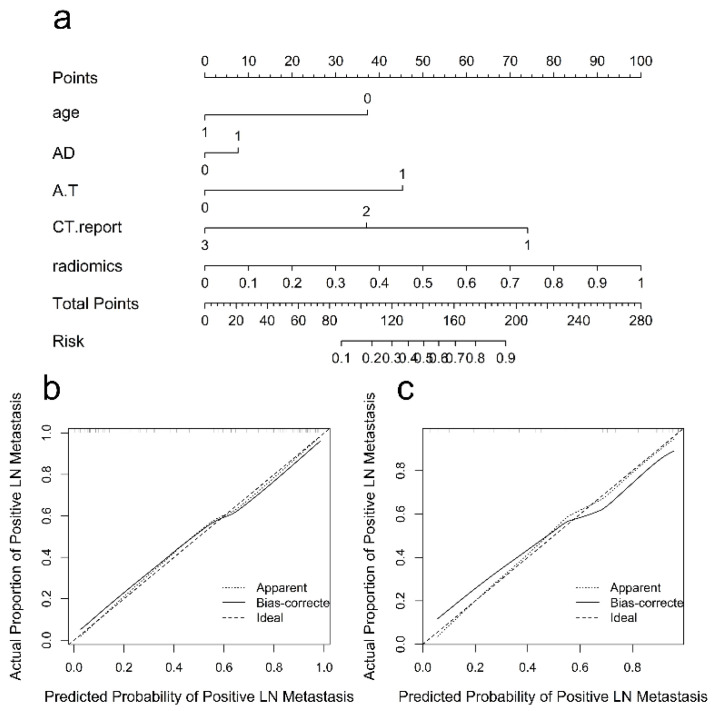
Comparison and calibration curves of the nomograms of the combined model. (**a**) A radiomics nomogram of the combined model incorporating age, AD, A/T, CT-reported lymph node status and radiomics signature. (**b**) Calibration curve of the nomogram of the combined model in the training cohort. The Hosmer–Lemeshow test yielded a nonsignificant statistic (*p* = 0.454). (**c**) Calibration curve of the nomogram of the combined model in the test cohort (*p* = 0.248). Calibration curves describe the model’s calibration in terms of agreement between the predicted probability of LNM and the actual lymph node status. The dotted line represents perfect performance, the gray solid line represents the actual performance and the black solid line represents the corrected prediction performance of the nomogram of the combined model. Abbreviations: LNM, lymph node metastasis; AD, anteroposterior diameter; A/T, anteroposterior to transverse diameter ratio; CT, computed tomography.

**Figure 5:**
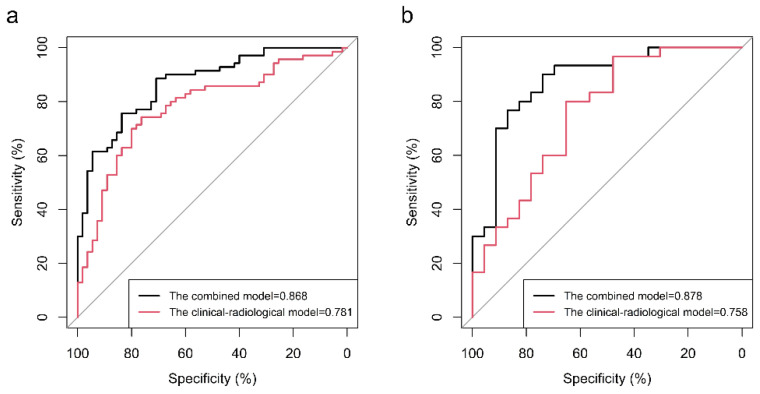
Comparison of the receiver operating characteristic (ROC) curves of the clinical–radiological model and the combined model in the training cohort (**a**) and test cohort (**b**). The predictive performance of the combined model was better than that of the clinical–radiological model in both the training and the test cohort. Abbreviation: ROC, receiver operating characteristic.

**Figure 6:**
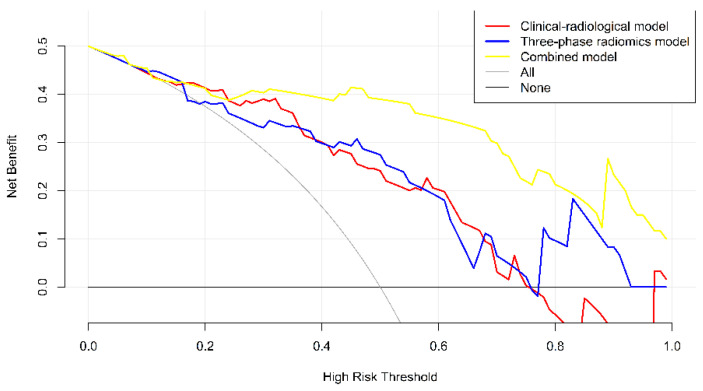
Decision curve analysis for the nomograms of the clinical–radiological and combined models. The black line represents the assumption that no patients have LNM. The gray line represents the assumption that all patients have LNM. The red line represents the net benefit at different threshold probabilities of the clinical–radiological model. The blue line represents the net benefit at different threshold probabilities of the three-phase radiomics model. The yellow line represents the net benefit at different threshold probabilities of the combined model. Among the three models, the combined model showed the highest overall net benefit. Abbreviations: LNM, lymph node metastasis; PTC, papillary thyroid carcinoma.

The radiomics signature, age, AD, A/T and CT-reported LN status 1 were identified as independent predictive factors for LNM by multivariable logistic regression analysis to construct the combined model. A radiomics nomogram was developed by integrating the radiomics signature and clinical–radiological factors (Figure 4a). The calibration curves of the radiomics nomogram showed good agreement between the predicted LNM probabilities and the actual pathological findings in both the training cohort (Figure 4b) and test cohort (Figure 4c), and the Hosmer–Lemeshow test yielded *p*-values of 0.454 and 0.248, respectively. In the training cohort, the combined nomogram integrating clinical–radiological factors and the radiomics signature had a significantly better predictive performance than the best clinical–radiological model (AUC, 0.868 vs. 0.781, DeLong test *p* = 0.003; ACC, 86.83% vs. 75.20%) (Figure 5a). In the test cohort, the combined nomogram also showed better performance than the clinical–radiological model (AUC, 0.878 vs. 0.758, DeLong test *p* = 0.017; ACC, 83.02% vs. 73.58%) (Figure 5b). The decision curve analysis for the combined nomogram, the three-phase radiomics model and the clinical–radiological model are illustrated in Figure 6. Among the three methods, the combined model showed a higher overall net benefit than the other two models. When the threshold probability was between 0.1 and 0.18, using the nomogram based only on the three-phase radiomics model offered a higher net benefit than treating all patients or treating no patients. When the threshold probability was between 0.18 and 0.25, a higher net benefit was obtained by using only the nomogram based on the clinical–radiological model. The combined nomogram exhibited a greater net benefit in predicting LNM than the three-phase radiomics model and the clinical–radiological model for a threshold probability higher than 24%.

## 4. Discussion

In our study, a comprehensive analysis integrating CT radiomic features, clinical factors and radiological characteristics was performed to preoperatively predict the LNM status of PTC patients. The results showed that CT radiomics features provided a similar discriminative value to clinical–radiological factors in both the training and test cohorts. However, the combined model integrating the clinical–radiological factors and radiomics features had a significantly improved predictive performance. Finally, a combined nomogram was established for individualized predicting of LNM probability in PTC, showing good agreement in the calibration and the best net benefit in the decision curve analysis. Our findings support the incorporation of radiomics analysis into the clinical workflow to improve the preoperative diagnosis of LNM in patients with PTC.

At present, although there is controversy in clinical practice about the possible impact of the radiation dose caused by the use of enhanced CT, according to the American Thyroid Association guidelines, CT is an effective adjunct imaging modality to US for patients with clinically suspected disease progression, such as aggressive primary tumor or enlarged LNM [7,44]. Several previous studies suggest that it is not necessary to delay radioactive iodine therapy when concerned about the excessive iodine content of contrast-enhanced CT, because the iodine clears up in 4–8 weeks [45,46]. Therefore, recent guidelines and studies suggest CT for the detection of metastatic lymph nodes in patients with PTC [47,48]. However, routine clinical US and CT diagnoses are greatly affected by the clinical experience and subjectivity of the radiologist. We hope to create a more objective and comprehensive method to predict LNM in PTC patients.

To study the efficacy of routine CT diagnosis combined with clinical risk factors in predicting LNM in PTC patients, we retrospectively collected 18 clinical factors and combined them with three CT-reported LN statuses diagnosed by three radiologists with 15, 7 and 5 years of diagnostic experience in head and neck cancers to build three clinical–radiological models. Chi-square tests showed that there were significant differences in age, capsule, location, AD, TD, A/T, CT-reported LN status 1 and CT-reported LN status 3 between the metastatic group and the non-metastatic group. Hîțu et al. found that the total tumor diameter and unilateral multifocality were independent predicting factors of metastatic papillary thyroid microcarcinoma [49]. Our study shows roughly the same results, that AD and TD were the independent radiological risk factors for diagnosing LNM of PTC. Since patients with multifocality were excluded in our study, whether unilateral multifocality is an independent predictor factor needs further verification. To explore the efficacy of radiologists with different working experience in diagnosing cervical LNM in patients with PTC, CT-reported LN status 2 was also included in the clinical–radiological model 2. In the test cohort, the clinical–radiological model 1 showed the best performance with AUC of 0.758, ACC of 73.58%, sensitivity of 80.00% and relatively low specificity of 65.22%. The sensitivities of the three clinical–radiological models were relatively higher (73.33–80.00%), the specificities were lower (65.22–69.57%) and the accuracy was from 69.81% to 73.58%. Despite combining the clinical risk factors with the diagnostic result of 15 years of diagnostic experience, the clinical–radiological model had a relatively lower diagnostic efficacy in predicting LNM in PTC patients.

For the radiomics model, the high-throughput feature extraction and selection is the key to radiomics model construction. These high-dimensional imaging features cannot be directly recognized by the naked eye. High-throughput feature extraction and selection captures the heterogeneity in the lesions in a non-invasive way, quantifies the deep-seated features and forms a potential database. Then, we can select the features with the most tumor information and analyze the relationship between these features and the LNM results by using the machine learning method of multiple logistic regression analysis to build the predictive radiomics model. In this study, we built four radiomics models: noncontrast model, arterial contrast model, venous contrast model and three-phase radiomics model. PCC or PCA dimensionality reduction method and multiple logistic regression analysis were used to establish these models. Nine of the fourteen radiomics features that constructed the three-phase radiomics model belong to shape features, such as the major axis length, minor axis length, sphericity, surface volume ratio and maximum 2D diameter row. This may indicate that the shape of the tumor is closely related to LNM of PTC patients. There were four radiomics features belonging to gray-level size zone matrix (GLSZM) texture features and one belonging to gray-level cooccurrence matrix (GLCM) texture features after the wavelet transform of the original CT images. These texture features represented noise removal and edge enhancement, and the radiomics signature combining these features from three-phase images describing different aspects of tumor appearance might capture hidden characteristics, offer insight into the heterogeneity of the tumor microenvironment (calcification, bleeding, cystic change, etc.) and thus create a more accurate model to predict the LNM of PTC patients. The diagnostic performances of the four radiomics models were close. Although the radiomic features of the three phase images were combined, no significant improvement in the diagnostic performance of the three-phase radiomics model was found. In the test cohort, the sensitivities of the noncontrast model, venous contrast model and three-phase radiomics model were relatively higher (80.00–86.67%), the specificities were relatively lower (60.87–78.26%), the accuracies were from 73.58% to 79.25% and the AUCs were from 0.781 to 0.813. The arterial contrast model had higher specificity (86.96%) but lower sensitivity (66.67%), although the Delong test found that the diagnostic performance of the radiomics model was similar to that of the clinical–radiological model. The radiomics method based on the computer image processing and machine learning modeling has some special advantages for the prediction of LNM in PTC patients, such as being more objective, more automated and less time-consuming.

Radiomics nomograms provide physicians with a visual and quantitative tool to identify LNM in patients with PTC and present recommendations/guidance for clinical decision making [29,50]. Lu et al. proposed a radiomics nomogram that incorporated radiomics features, CT-reported LN status, sex and age for the preoperative prediction of cervical LN metastasis in patients with PTC [37]. Zhou et al. proposed a radiomics nomogram that incorporated conventional CT images with radiomics features dual-energy CT-derived iodine maps in diagnosing cervical LNM in patients with PTC [38]. These two successful studies indicated the feasibility of applying radiomics to predict LN status in PTC. In our study, when the most diagnostic clinical factors, radiological characteristics and radiomics features were integrated, the combined nomogram yielded the best predictive performances with an AUC, ACC, sensitivity and specificity of 0.868, 86.83%, 88.57% and 70.91%, respectively, in the training cohort and 0.878, 83.02%, 90.00% and 73.91%, respectively, in the test cohort. Compared with the previous CT radiomics (AUC = 0.822 in the test cohort) [37] and ultrasound radiomics (AUC = 0.727 in the test cohort) [36] research for the prediction of LNM in PTC patients, our model achieved good diagnostic efficacy. Decision curve analysis was used to demonstrate the potential application value of our nomogram; this analysis confirmed the increased value of our research method combining radiomics features and clinical risk predictors for the prediction of LNM in PTC through the net benefit according to the threshold probability. Although the combined model has good diagnostic efficacy in preoperative prediction of LNM of PTC patients, this study is not intended to replace the radiologist’s diagnosis. We focus more on exploring the possibility of embedding radiomics technologies based on computer image processing into clinical workflows, and identifying the increased value of integrating CT radiomics signature with radiologist diagnosis and clinical factors (age, AD and A/T) to construct a combined model for the accurate prediction of LNM of PTC patients. Our proposed combined nomogram can give radiologists more confidence to make accurate diagnoses for LNM states and give surgeons more confidence to develop optimal treatment strategies and assess the prognosis of PTC patients.

Although the proposed comprehensive radiomics analysis has certain advantages over conventional clinical and radiological factors, the limitations of our study merit discussion. First, our model was trained and tested based on retrospectively collected datasets from a single center, and the reproducibility and robustness of the model must be externally validated through multicenter and prospective studies. Second, the heterogeneity of the imaging parameters between two different CT machines may have a certain impact on the results. Previous studies have shown that CT radiomics features could be affected by slice thickness, bin width, voxel size, number of gray levels, etc., in images from different CT scanners [51]. Appropriate image preprocessing, such as voxel size normalization and gray level normalization, can reduce the impact of image parameter changes on the variability of radiomic features [52]. In our study, although all CT images were normalized to 0–256 gray levels before feature extraction, the voxel size normalization was not applied to the CT images. We think this may affect the stability of the texture features, and thus the prediction efficiency of the radiomics model. As the sample size of our dual-source CT cases increases, the extent of this impact will be investigated in future research. Third, radiomics analysis was only performed on conventional and enhanced CT images. In future studies, we will incorporate iodine maps and other spectral images from dual-energy CT in radiomics analysis to mine valuable information and improve the predictive performance for LNM in PTC.

## 5. Conclusions

In conclusion, we developed a combined model based on CT imaging features and clinical risk factors to predict cervical LNM in patients with PTC. Radiomics analysis plays an important role in diagnosing cervical LNM in PTC, and our combined model performed better than the other models. The nomogram of the combined model based on preoperative CT can improve the accuracy of the prediction of cervical LNM in PTC patients and help clinicians make more reasonable clinical diagnosis and treatment decisions.

## Figures and Tables

**Table 1:** Associations between LNM and clinical factors.

Characteristics	LNM (+) (N = 100)	LNM (−) (N = 78)	*p*-Value
Age, mean ± SD, years	42.55 ± 14.28	49.45 ± 9.95	
<45 NO. (%)	56 (56.00)	24 (30.77)	0.001
≥45 NO. (%)	44 (44.00)	54 (69.23)	
Sex NO. (%)			
Male	28 (28.00)	15 (19.23)	0.175
Female	72 (72.00)	63 (80.77)	
BMI NO. (%)			
Normal	48 (48.00)	35 (44.87)	0.678
Abnormal	52 (52.00)	43 (55.13)	
TG NO. (%)			
Normal	66 (66.00)	56 (71.79)	0.409
Abnormal	34 (34.00)	22 (28.21)	
TGAb NO. (%)			
Normal	30 (30.00)	29 (37.18)	0.313
Abnormal	70 (70.00)	49 (62.82)	
TPOAb NO. (%)			
Normal	51 (51.00)	37 (47.44)	0.637
Abnormal	49 (49.00)	41 (52.56)	
FT3 NO. (%)			
Normal	71 (71.00)	64 (82.05)	0.087
Abnormal	29 (29.00)	14 (17.95)	
FT4 NO. (%)			
Normal	45 (45.00)	33 (42.31)	0.719
Abnormal	55 (55.00)	45 (57.69)	
TSH NO. (%)			
Normal	58 (58.00)	50 (64.10)	0.408
Abnormal	42 (42.00)	28 (35.90)	
AD NO. (%)			
<6 mm	4 (4.00)	22 (28.21)	<0.001
≥6 mm	96 (96.00)	56 (71.79)	
TD NO. (%)			
<6 mm	10 (10.00)	27 (34.62)	<0.001
≥6 mm	90 (90.00)	51 (65.38)	
A/T NO. (%)			
<1	15 (15.00)	28 (35.90)	0.001
≥1	85 (85.00)	50 (64.10)	
HT NO. (%)			
Not involved	94 (94.00)	75 (96.15)	0.515
Involved	6 (6.00)	3 (3.85)	
NG NO. (%)			
Not involved	70 (70.00)	62 (79.49)	0.151
Involved	30 (30.00)	16 (20.51)	
Capsule NO. (%)			
Not involved	36 (36.00)	41 (52.56)	0.027
Involved	64 (64.00)	37 (47.44)	
Calcification NO. (%)			
Negative	48 (48.00)	45 (57.69)	0.199
Positive	52 (52.00)	33 (42.31)	
Location NO. (%)			
Left lobe	52 (52.00)	32 (41.03)	
Isthmus	0 (0.00)	6 (7.69)	0.012
Right lobe	48 (48.00)	40 (51.28)	
CT reported-lymph node status 1 NO. (%)			
LNM-positive	61 (61.00)	15 (19.23)	
LNM-suspicious	23 (23.00)	17 (21.79)	<0.001
LNM-negative	16 (16.00)	46 (58.97)	
CT reported-lymph node status 2 NO. (%)			
LNM-positive	59 (59.00)	35 (44.87)	
LNM-suspicious	18 (18.00)	12 (15.38)	0.053
LNM-negative	23 (23.00)	31 (39.74)	
CT reported-lymph node status 3 NO. (%)			
LNM-positive	58 (58.00)	27 (34.62)	
LNM-suspicious	13 (13.00)	4 (5.13)	<0.001
LNM-negative	29 (29.00)	47 (60.26)	

There were significant differences in age, capsule, location, AD, TD, A/T, CT-reported lymph node status 1 and CT-reported lymph node status 3 between the two groups (*p* < 0.05), with no differences in the other clinical factors (*p* > 0.05). Abbreviations: LNM, lymph node metastasis; BMI, body mass index; TG, thyroglobulin; TGAb, thyroglobulin antibody; TPOAb, thyroid peroxidase antibody; FT3, free triiodothyronine; FT4, free thyroxine; TSH, thyroid-stimulating hormone; AD, anteroposterior diameter; TD, transverse diameter; A/T, anteroposterior to transverse diameter ratio; HT, Hashimoto’s thyroiditis; NG, nodular goiter; CT, computed tomography.

**Table 2:** Clinical characteristics of the patients in the training and test cohorts.

Characteristics	Training Cohort (N = 125)		*p*-Value	Test Cohort (N = 53)		*p*-Value
	LNM (+) (N = 70)	LNM (−) (N = 55)		LNM (+) (N = 30)	LNM (−) (N = 23)	
Age, mean ± SD, years	42.64 ± 13.927	49.15 ± 9.519		42.33 ± 15.320	50.17 ± 11.092	
<45 NO. (%)	42 (60.00)	17 (30.91)	0.001	14 (46.67)	7 (30.43)	0.231
≥45 NO. (%)	28 (40.00)	38 (69.09)		16 (53.33)	16 (69.57)	
Sex NO. (%)						
Male	21 (30.00)	10 (18.18)	0.129	7 (23.33)	5 (21.74)	0.891
Female	49 (70.00)	45 (81.82)		23 (76.67)	18 (78.26)	
BMI NO. (%)						
Normal	34 (48.57)	26 (47.27)	0.885	14 (46.67)	9 (39.13)	0.583
Abnormal	36 (51.43)	29 (52.73)		16 (53.33)	14 (60.87)	
TG NO. (%)						
Normal	46 (65.71)	41 (74.55)	0.287	20 (45.83)	15 (65.22)	0.912
Abnormal	24 (34.29)	14 (25.45)		10 (54.17)	8 (34.78)	
TGAb NO. (%)						
Normal	22 (31.43)	20 (36.36)	0.562	8 (26.67)	9 (39.13)	0.335
Abnormal	48 (68.57)	35 (63.64)		22 (73.33)	14 (60.87)	
TPOAb NO. (%)						
Normal	36 (40.00)	26 (41.67)	0.645	15 (50.00)	11 (47.83)	0.875
Abnormal	34 (60.00)	29 (58.33)		15 (50.00)	12 (52.17)	
FT3 NO. (%)						
Normal	48 (68.57)	45 (81.82)	0.092	23 (76.67)	19 (82.61)	0.597
Abnormal	22 (31.43)	10 (18.18)		7 (23.33)	4 (17.39)	
FT4 NO. (%)						
Normal	30 (42.86)	23 (41.82)	0.907	15 (50.00)	10 (43.48)	0.637
Abnormal	40 (57.14)	32 (58.18)		15 (50.00)	13 (56.52)	
TSH NO. (%)						
Normal	42 (60.00)	36 (65.45)	0.532	16 (53.33)	14 (60.87)	0.583
Abnormal	28 (40.00)	19 (34.55)		14 (46.67)	9 (39.13)	
AD NO. (%)						
<6 mm	3 (4.29)	12 (21.82)	0.003	1 (3.33)	10 (43.48)	<0.001
≥6 mm	67 (95.71)	43 (78.18)		29 (96.67)	13 (56.52)	
TD NO. (%)						
<6 mm	9 (12.86)	18 (32.73)	0.007	1 (3.33)	9 (39.13)	0.001
≥6 mm	61 (87.14)	37 (67.27)		29 (76.67)	14 (60.87)	
A/T NO. (%)						
<1	8 (11.43)	18 (32.73)	0.004	7 (23.33)	10 (43.48)	0.119
≥1	62 (88.57)	37 (67.27)		23 (76.67)	13 (56.52)	
HT NO. (%)						
Not involved	65 (92.86)	53 (96.36)	0.397	29 (96.67)	22 (95.65)	0.848
Involved	5 (7.14)	2 (3.64)		1 (3.33)	1 (4.35)	
NG NO. (%)						
Not involved	49 (70.00)	43 (78.18)	0.303	21 (70.00)	19 (82.61)	0.290
Involved	21 (30.00)	12 (21.82)		9 (30.00)	4 (17.39)	
Capsule NO. (%)						
Not involved	25 (35.71)	26 (47.27)	0.192	11 (36.67)	15 (65.22)	0.039
Involved	45 (64.29)	29 (52.73)		19 (63.33)	8 (34.78)	
Calcification NO. (%)						
Negative	35 (50.00)	34 (61.82)	0.187	13 (43.33)	11 (47.83)	0.745
Positive	35 (50.00)	21 (38.18)		17 (56.67)	12 (52.17)	
Location NO. (%)						
Left lobe	34 (48.57)	22 (40.00)		18 (60.00)	10 (43.48)	
Isthmus	0 (0.00)	5 (9.09)	0.032	0 (0.00)	1 (4.35)	0.301
Right lobe	36 (51.43)	28 (50.91)		12 (40.00)	12 (52.17)	
CT reported-lymph node status 1 NO. (%)						
LNM-positive	43 (61.43)	9 (16.36)		18 (60.00)	6 (26.09)	
LNM-suspicious	17 (24.29)	12 (21.82)	<0.001	6 (20.00)	5 (21.74)	0.026
LNM-negative	10 (14.29)	34 (61.82)		6 (20.00)	12 (52.17)	
CT reported-lymph node status 2 NO. (%)						
LNM-positive	44 (62.86)	24 (43.64)		15 (50.00)	11 (47.83)	
LNM-suspicious	10 (14.29)	8 (14.55)	0.06	8 (26.67)	4 (17.39)	0.574
LNM-negative	16 (22.86)	23 (41.82)		7 (23.33)	8 (34.78)	
CT reported-lymph node status 3 NO. (%)						
LNM-positive	39 (55.71)	17 (30.91)		19 (63.33)	10 (43.48)	
LNM-suspicious	9 (12.86)	3 (5.45)	0.002	4 (13.33)	1 (4.35)	0.079
LNM-negative	22 (40.00)	35 (63.64)		7 (23.33)	12 (52.17)	

There were significant differences in age, location, AD, TD, A/T, CT-reported lymph node status 1 and CT-reported lymph node status 3 between patients with and without LNM in the training cohort (*p* < 0.05), with no differences in the other clinical factors (*p* > 0.05). However, in the test cohort, only AD, TD, capsule and CT-reported lymph node status 1 were significantly different between patients with and without LNM (*p* < 0.05); the other clinical factors were not significantly different between groups (*p* > 0.05). Abbreviations: LNM, lymph node metastasis; BMI, body mass index; TG, thyroglobulin; TGAb, thyroglobulin antibody; TPOAb, thyroid peroxidase antibody; FT3, free triiodothyronine; FT4, free thyroxine; TSH, thyroid-stimulating hormone; AD, anteroposterior diameter; TD, transverse diameter; A/T, anteroposterior to transverse diameter ratio; HT, Hashimoto’s thyroiditis; NG, nodular goiter; CT, computed tomography.

**Table 3:** Associations between actual lymph node status, clinical characteristics and CT-reported status.

Characteristics	Training Cohort (N = 125)	Test Cohort (N = 53)	*p*-Value
Age, mean ± SD, years	45.50 ± 12.566	45.74 ± 14.084	
<45 NO. (%)	59 (47.20)	21 (39.62)	0.353
≥45 NO. (%)	66 (52.80)	32 (60.38)	
Sex NO. (%)			
Male	31 (24.80)	12 (22.64)	0.758
Female	94 (75.20)	41 (77.36)	
BMI NO. (%)			
Normal	60 (48.00)	23 (43.40)	0.573
Abnormal	65 (52.00)	30 (56.60)	
TG NO. (%)			
Normal	87 (69.60)	35 (66.04)	0.640
Abnormal	38 (30.40)	18 (33.94)	
TGAb NO. (%)			
Normal	42 (33.60)	17 (32.08)	0.843
Abnormal	83 (66.40)	36 (67.92)	
TPOAb NO. (%)			
Normal	62 (49.60)	26 (49.06)	0.947
Abnormal	63 (50.40)	27 (50.94)	
FT3 NO. (%)			
Normal	93 (74.40)	42 (79.25)	0.490
Abnormal	32 (25.60)	11 (20.75)	
FT4 NO. (%)			
Normal	53 (42.40)	25 (47.17)	0.558
Abnormal	72 (57.60)	28 (52.83)	
TSH NO. (%)			
Normal	78 (62.40)	30 (56.60)	0.469
Abnormal	47 (37.60)	23 (43.40)	
AD NO. (%)			
<6 mm	15 (12.00)	11 (20.75)	0.130
≥6 mm	110 (88.00)	42 (79.25)	
TD NO. (%)			
<6 mm	27 (21.60)	10 (18.87)	0.681
≥6 mm	98 (78.40)	43 (81.13)	
A/T NO. (%)			
<1	26 (20.80)	17 (32.08)	0.108
≥1	99 (79.20)	36 (67.92)	
HT NO. (%)			
Not involved	118 (94.40)	51 (96.23)	0.611
Involved	7 (5.60)	2 (3.77)	
NG NO. (%)			
Not involved	92 (73.60)	40 (75.47)	0.794
Involved	33 (26.40)	13 (24.53)	
Capsule NO. (%)			
Not involved	51 (40.80)	26 (49.06)	0.309
Involved	74 (59.20)	27 (50.94)	
Calcification NO. (%)			
Negative	69 (55.20)	24 (45.28)	0.226
Positive	56 (44.80)	29 (54.72)	
Location NO. (%)			
Left lobe	56 (44.80)	28 (52.83)	
Isthmus	5 (4.00)	1 (1.89)	0.531
Right lobe	64 (51.20)	24 (45.28)	
CT reported-lymph node status1 NO. (%)			
LNM-positive	52 (41.60)	24 (45.28)	
LNM-suspicious	29 (23.20)	11 (20.75)	0.890
LNM-negative	44 (35.20)	18 (33.96)	
CT reported-lymph node status 2 NO. (%)			
LNM-positive	68 (54.40)	26 (49.06)	
LNM-suspicious	18 (14.40)	12 (22.64)	0.406
LNM-negative	39 (31.20)	15 (28.30)	
CT reported-lymph node status 3 NO. (%)			
LNM-positive	56 (44.80)	29 (54.72)	
LNM-suspicious	12 (9.60)	5 (9.43)	0.450
LNM-negative	57 (45.60)	19 (35.85)	
LNM status NO. (%)			
Negative	70 (56.00)	30 (56.60)	0.941
Positive	55 (44.00)	23 (43.40)	

No significant differences were found between the two cohorts in terms of age, sex, BMI, TG, TGAb, TPOAb, FT3, FT4, TSH, AD, TD, A/T, HT, NG, capsule, calcification, location, CT-reported lymph node status or LNM status (*p* > 0.05). Abbreviations: LNM, lymph node metastasis; BMI, body mass index; TG, thyroglobulin; TGAb, thyroglobulin antibody; TPOAb, thyroid peroxidase antibody; FT3, free triiodo-thyronine; FT4, free thyroxine; TSH, thyroid-stimulating hormone; AD, anteroposterior diameter; TD, transverse diameter; A/T, anteroposterior to transverse diameter ratio; HT, Hashimoto’s thyroiditis; NG, nodular goiter; CT, computed tomography.

**Table 4:** Diagnostic performance of the models.

Model Categories	Training					Test				
	Sensitivity	Specificity	Accuracy	AUC	*p*-Value	Sensitivity	Specificity	Accuracy	AUC	*p*-Value
Clinical–radiological model 1	74.29	76.36	75.20	0.781	0.003	80.00	65.22	73.58	0.758	0.017
Clinical–radiological model 2	85.71	63.64	76.00	0.796	0.036	73.33	69.57	71.70	0.729	0.024
Clinical–radiological model 3	74.29	78.18	76.00	0.800	0.045	73.33	65.22	69.81	0.743	0.052
Noncontrast model	84.29	58.18	72.80	0.786	0.068	80.00	65.22	73.58	0.781	0.141
Arterial contrast model	71.43	78.18	74.40	0.808	0.212	66.67	86.96	75.47	0.791	0.296
Venous contrast model	87.14	63.64	76.80	0.827	0.343	86.67	60.87	75.47	0.790	0.224
Three-phase radiomics model	78.57	65.45	72.80	0.790	0.011	80.00	78.26	79.25	0.813	0.116
Combined model	88.57	70.91	86.83	0.868	--	90.00	73.91	83.02	0.878	--

The LNM status in clinical–radiological model 1, clinical–radiological model 2 and clinical–radiological model 3 was determined by radiologists who had been engaged in head and neck imaging diagnosis for 15 years, 7 years and 5 years, respectively. Abbreviations: LNM, lymph node metastasis; AUC, area under the curve; *p*-value, comparison of diagnostic performance between combined model and other models.

**Table 5:** Diagnostic performance of the combined model in different LN location subgroups.

LNM Location Categories	Sensitivity	Specificity	Accuracy	PPV	NPV	AUC
Central LNM prediction	78.79	72.73	75.58	74.29	77.42	0.833
Lateral LNM prediction	66.67	81.48	74.07	78.26	70.97	0.823
Central and lateral LNM prediction	87.50	85.50	86.25	85.37	87.18	0.960

Abbreviations: LNM, lymph node metastasis; PPV, positive predictive value; NPV, negative predictive value; AUC, area under the curve.

## Data Availability

The datasets used and/or analyzed during the current study are available from the corresponding author upon reasonable request.

## Supplementary Materials

The following supporting information can be downloaded at: https://www.mdpi.com/article/10.3390/diagnostics12051119/s1, Table S1: The coefficients of features in the clinical–radiological model; Table S2: The coefficients of radiomics features in the different models.
