# Supplementary material for: Radiomics Profiling Identifies the Value of CT Features for the Preoperative Evaluation of Lymph Node Metastasis in Papillary Thyroid Carcinoma
Source: Diagnostics (Basel). 2022 Apr 29;12(5):1119. doi: 10.3390/diagnostics12051119 (PMC9139816; doi:10.3390/diagnostics12051119)
Supplement: Supplementary file 1 [file diagnostics-12-01119-s001.zip › diagnostics-1665239-supplementary.pdf]

Table S1. The coefficients of features in the clinical-radiological model

Model categories	Features	Coefficients
Clinical-radiological model 1	PCA_feature_1	0.650
	PCA_feature_5	-0.783
Clinical-radiological model 2	Age	-4.101
	CT-report	-1.245
	AD	-0.510
	TD	5.688
	A/T	2.240
	Capsule	0.819
	Location	0.111
Clinical-radiological model 3	PCA_feature_1	0.443
	PCA_feature_2	-0.803
	PCA_feature_3	0.074
	PCA_feature_4	0.293
	PCA_feature_5	0.108
	PCA_feature_6	-0.075

Table S2. The coefficients of radiomics features in the different models

Model categories	Features	Coefficients
Noncontrast model	NC_original_ngtdm_Coarseness	-2.959
	NC _original_shape_LeastAxisLength	-3.272
	NC _original_shape_MajorAxisLength	6.304
	NC _original_shape_Maximum2DDiameterRow	2.473
	NC _original_shape_Maximum2DDiameterSlice	-0.542
	NC _original_shape_MinorAxisLength	-1.077
	NC _original_shape_SurfaceVolumeRatio	-1.040
	NC _wavelet-HHH_ngtdm_Coarseness	1.661
	NC _wavelet-HLH_glszm_ZoneEntropy	-0.292
	NC _wavelet-HLL_glszm_ZoneEntropy	3.384
	NC _wavelet-HLL_ngtdm_Coarseness	-1.373
	NC_wavelet-LLH_gldm_SmallDependenceLowGrayLevelEmphasis	-0.758
	NC _wavelet-LLL_glcm_Idn	0.798
	NC_wavelet-LLL_gldm_SmallDependenceLowGrayLevelEmphasis	0.719
	NC _wavelet-LLL_glszm_ZoneEntropy	-4.093
	NC _wavelet-LLL_ngtdm_Coarseness	0.029
Arterial contrast model	A_original_shape_LeastAxisLength	-2.768
	A_original_shape_MajorAxisLength	5.493
	A_original_shape_Maximum2DDiameterRow	4.552
	A_original_shape_Maximum2DDiameterSlice	-3.203
	A_original_shape_MinorAxisLength	-1.775
	A_original_shape_Sphericity	-1.274
	A_original_shape_SurfaceVolumeRatio	-0.617

	A_wavelet-HHH_glcm_MaximumProbability	-1.679
	A_wavelet-HHH_gldm_LargeDependenceEmphasis	1.068
	A_wavelet-HHH_gldm_LargeDependenceLowGrayLevelEmphasis	-2.052
	A_wavelet-HHH_glrlm_LongRunLowGrayLevelEmphasis	-1.541
	A_wavelet-HHL_glszm_ZonePercentage	-3.954
	A_wavelet-HLH_glszm_ZoneEntropy	3.221
	A_wavelet-LLL_glcm_Idn	-2.188
	A_wavelet-LLL_gldm_SmallDependenceLowGrayLevelEmphasis	-0.628
	V_original_shape_LeastAxisLength	-4.439
	V_original_shape_MajorAxisLength	3.697
	V_original_shape_Maximum2DDiameterRow	3.138
	V_original_shape_Maximum2DDiameterSlice	-6.573
	V_original_shape_MinorAxisLength	1.433
Venous contrast model	V_original_shape_Sphericity	-3.090
	V_original_shape_SurfaceArea	2.766
	V_original_shape_SurfaceVolumeRatio	-1.158
	V_wavelet-HLH_glszm_ZoneEntropy	0.126
	V_wavelet-LHH_glcm_Id	-6.108
	V_wavelet-LHH_glszm_ZoneEntropy	0.904
	A_original_shape_MajorAxisLength	0.214
	A_wavelet-HLH_glszm_ZoneEntropy	0.101
	A_original_shape_MinorAxisLength	-0.465
	V_original_shape_Sphericity	-0.294
	V_original_shape_Maximum2DDiameterRow	0.238
	V_wavelet-LHH_glszm_ZoneEntropy	0.074
Three-phase radiomics	V_wavelet-LHH_glcm_Id	-0.867
model	NC _original_shape_MajorAxisLength	0.758
	NC _original_shape_Maximum2DDiameterRow	0.049
	NC _original_shape_SurfaceVolumeRatio	-0.154
	NC _original_shape_MinorAxisLength	0.055
	NC _original_shape_LeastAxisLength	-0.509
	NC _wavelet-HLL_glszm_ZoneEntropy	0.464
	NC _wavelet-HLH_glszm_ZoneEntropy	-0.302
	PCA_feature_1	0.406
	PCA_feature_2	0.498
	PCA_feature_3	-0.773
	PCA_feature_4	0.400
	PCA_feature_5	-0.814
Combined model	PCA_feature_6	0.246
	PCA_feature_8	-0.534
	PCA_feature_9	-0.481
	PCA_feature_11	0.608
	PCA_feature_16	-0.414
